# Supplementary material for: Splice-Junction-Based Mapping of Alternative Isoforms in the Human Proteome
Source: Cell Rep. Author manuscript; Available in PMC 2020 Jan 15. (PMC6961840; doi:10.1016/j.celrep.2019.11.026)
Supplement: 2 [file NIHMS1546469-supplement-2.zip › DF1/PXD010154/Testis/Testis_2_FBLN2_CEDIDECAQGAGILCTFR.pdf]

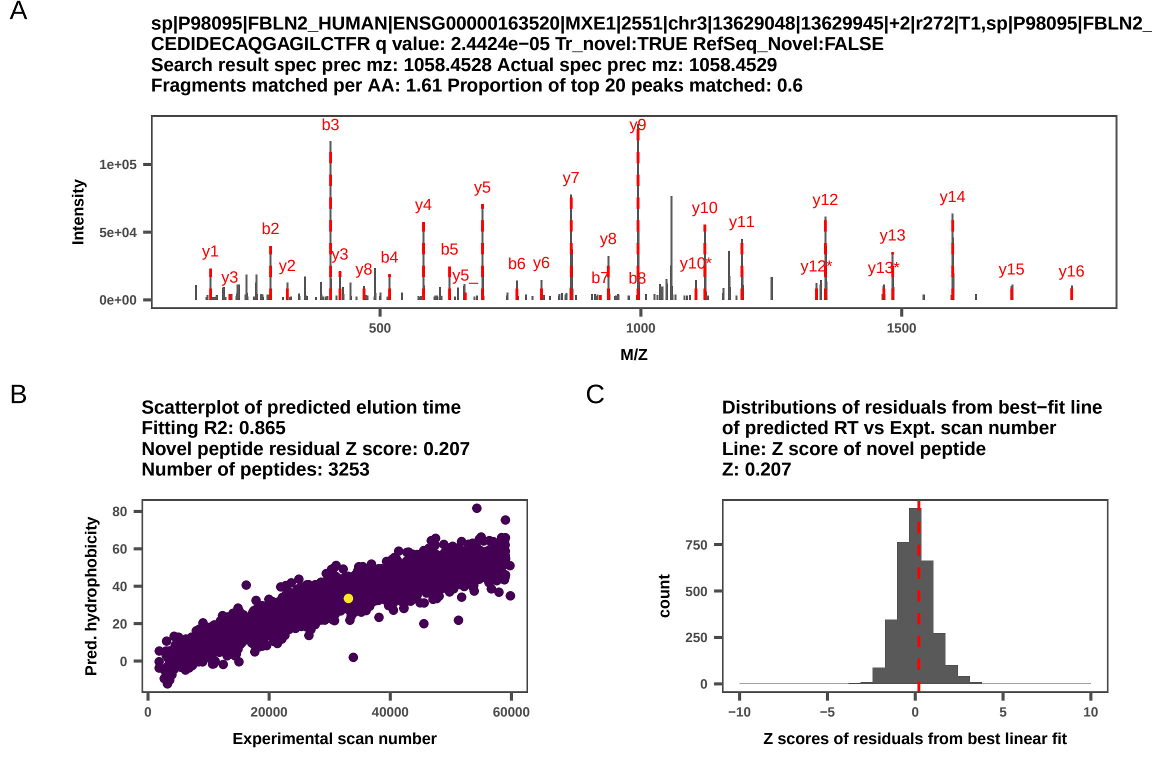

A
sp|P98095|FBLN2_HUMAN|ENSG00000163520|MXE1|2551|chr3|13629048|13629945|+2|r272|T1,sp|P98095|FBLN2_
CEDIDECAQGAGILCTFR q value: 2.4424e−05 Tr_novel:TRUE RefSeq_Novel:FALSE
Search result spec prec mz: 1058.4528 Actual spec prec mz: 1058.4529
Fragments matched per AA: 1.61 Proportion of top 20 peaks matched: 0.6
Intensity
1e+05
5e+04
0e+00
y1
y3
b2
y2
b3
y3
y8
b4
y4
b5
y5_
y5
b6
y6
y7
b7
y8
y9
b8
y10*
y10
y11
y12*
y12
y13*
y13
y14
y15
y16
500
1000
1500
M/Z
B
Scatterplot of predicted elution time
Fitting R2: 0.865
Novel peptide residual Z score: 0.207
Number of peptides: 3253
Pred. hydrophobicity
80
60
40
20
0
0
20000
40000
60000
Experimental scan number
C
Distributions of residuals from best−fit line
of predicted RT vs Expt. scan number
Line: Z score of novel peptide
Z: 0.207
count
750
500
250
0
−10
−5
0
5
10
Z scores of residuals from best linear fit
